# Weaning in der neurologisch-neurochirurgischen Frührehabilitation – Ergebnisse der „WennFrüh“-Studie der Deutschen Gesellschaft für Neurorehabilitation

**DOI:** 10.1007/s00115-020-00976-z

**Published:** 2020-08-10

**Authors:** Jens D. Rollnik, Jan Brocke, Anna Gorsler, Martin Groß, Michael Hartwich, Marcus Pohl, Tobias Schmidt-Wilcke, Thomas Platz

**Affiliations:** 1 Institut für neurorehabilitative Forschung (InFo), Assoziiertes Institut der Medizinischen Hochschule Hannover (MHH), BDH-Klinik Hessisch Oldendorf gGmbH, Hessisch Oldendorf, Deutschland; 2 Frührehabilitation & Neurointensivmedizin, Neurologisches Zentrum, Segeberger Kliniken GmbH, Bad Segeberg, Deutschland; 3 Fachkrankenhaus für Neurologische Frührehabilitation, Kliniken Beelitz GmbH, Beelitz, Deutschland; 4 Klinik für Neurologische Intensivmedizin und Frührehabilitation, Evangelisches Krankenhaus Oldenburg, Medizinischer Campus Universität Oldenburg, Oldenburg, Deutschland; 5 Akutklinik für Neurologische Frührehabilitation, Asklepios Schlossberg Klinik Bad König, Bad König, Deutschland; 6 Fachklinik für Neurologisch-Neurochirurgische Rehabilitation, VAMED Klinik Schloss Pulsnitz GmbH, Pulsnitz, Deutschland; 7 St. Mauritius Therapieklinik, Meerbusch, Deutschland; 8 Institut für Klinische Neurowissenschaften und Medizinische Psychologie, Universitätsklinikum Düsseldorf, Düsseldorf, Deutschland; 9 Institut für Neurorehabilitation und Evidenzbasierung, An-Institut der Universität Greifswald, BDH-Klinik Greifswald gGmbH, Karl-Liebknecht-Ring 26a, 17491 Greifswald, Deutschland; 10 AG Neurorehabilitation, Universitätsmedizin Greifswald, Greifswald, Deutschland

**Keywords:** Neurologisch-neurochirurgische Frührehabilitation, Neurorehabilitation Beatmungsentwöhnung, Weaning, Rehabilitation, Heimbeatmung, Early neurological and neurosurgical rehabilitation, Neurorehabilitation, Mechanical ventilation, Weaning, Rehabilitation, Home ventilation

## Abstract

Neurologisch-neurochirurgische Frührehabilitanden sind klinisch oft so schwer betroffen, dass sie neben der frührehabilitativen Behandlung auch von der mechanischen Beatmung entwöhnt werden müssen. In einer Umfrage der Deutschen Gesellschaft für Neurorehabilitation (DGNR) wurden neurologische Weaningzentren gebeten, Informationen zu Strukturmerkmalen ihrer Einrichtung, ihrer personellen und apparativen Ausstattung sowie basierend auf anonymen Daten zu Fallzahl und Behandlungsergebnis zur Verfügung zu stellen. Es nahmen 36 Weaningeinheiten aus 11 Bundesländern mit insgesamt 496 Betten teil. Von 2516 erfassten Weaningfällen im Jahr 2019 wurden 2097 (83,3 %) primär erfolgreich entwöhnt und nur 120 (4,8 %) mussten mit Heimbeatmung entlassen werden. Die Mortalität in dieser Stichprobe lag bei 11,0 % (*n* = 276). Die Erhebung zeigt, dass das prolongierte Weaning in der neurologisch-neurochirurgischen Frührehabilitation ein wichtiger und erfolgreicher Bestandteil der Versorgung schwerstkranker Patienten darstellt.

## Abstract (en)

Neurological and neurosurgical early rehabilitation patients are often so critically ill that they must be weaned from mechanical ventilation in addition to early rehabilitative treatment. The German Society for Neurorehabilitation (DGNR) carried out a survey and asked neurological weaning units to provide information on structural characteristics of the facility, including personnel and technical resources and the number of cases and outcome based on anonymous data. In total 36 weaning units from 11 federal states with a total of 496 beds participated in the survey. From 2516 weaning cases documented in 2019, 2097 (83.3%) could primarily be successfully weaned from mechanical ventilation and only 120 (4.8%) had to be discharged with home ventilation. The mortality in this sample was 11.0% (*n* = 276). The results of the survey demonstrate that prolonged weaning during early neurological and neurosurgical rehabilitation is an important and effective component of healthcare provision for critically ill patients in Germany.

## Hintergrund

Das Weaning von der mechanischen Beatmung stellt neben der Entwöhnung von der Trachealkanüle und dem damit verbundenen Dysphagiemanagement eine wichtige Aufgabe in der neurologisch-neurochirurgischen Frührehabilitation (NNFR) dar. Dabei gibt es durch die zugrunde liegenden Schädigungen des zentralen bzw. peripheren Nervensystems erhebliche Besonderheiten im prolongierten Weaning [1, 2], wenn man es mit dem Weaning bei pneumologischen Patienten [2] vergleicht. Die Patienten der NNFR leiden oft an multiplen und schwerwiegenden neurologischen Symptomen wie Bewusstseinsstörungen, Paresen, Dysphagie und reduzierten Schutzreflexen, sodass häufig noch nach Abschluss der Entwöhnung vom Respirator eine aufwendige Entwöhnung von der Trachealkanüle stattfinden muss. Des Weiteren besteht bei diesen Patienten das Risiko schwerer Komplikationen wie Hydrozephalus, ZNS-Infektion oder Status epilepticus. Der nichtinvasiven Beatmung (NIV) kommt bei diesem Patientengut – im Vergleich zu einer pneumologischen Klientel – wegen der regelhaft anzutreffenden Dysphagie ein deutlich niedrigerer Stellenwert zu [2, 3].

Es steht außer Frage, dass schwer kranke Patienten nach einer neurologischen oder neurochirurgischen Behandlung im Akutkrankenhaus einer nahtlos sich anschließenden und indikationsspezifischen Rehabilitation in der NNFR bedürfen. In einer Untersuchung mit 1716 Schlaganfallpatienten wurde beispielsweise gezeigt, dass ein früher Beginn der Rehabilitation innerhalb der ersten Woche nach dem Ereignis zu einem signifikant besseren Outcome führte als ein Beginn zwischen 2 und 4 Wochen [4]. Dieser und andere Befunde haben in der bereits zitierten S2k-Leitlinie zu folgender Empfehlung geführt: „Beatmete Patienten mit Erkrankungen des zentralen und/oder peripheren Nervensystems und/oder (neuro-)muskulären Erkrankungen sollten so früh wie möglich in eine neurologisch-neurochirurgische Frührehabilitationseinrichtung mit intensivmedizinischer und Weaningkompetenz verlegt werden.“ [2].

Die NNFR ist als „Phase B“ integraler Bestandteil des Phasenmodells der Bundesarbeitsgemeinschaft für Rehabilitation (BAR), das die Versorgung von Patienten mit schweren und schwersten Hirnschädigungen in Deutschland sicherstellt [5]. Dabei folgt die NNFR – leistungsrechtlich zumeist als Krankenhausbehandlung gemäß § 39 SGB V – auf die primäre Akutbehandlung (Phase A). Mit zunehmender Selbständigkeit schließen sich die Phasen C und D (Anschlussrehabilitation) als medizinische Rehabilitation (§ 40 SGB V) an [5]. Unter der Phase E versteht man nachgehende Rehabilitationsmaßnahmen, z. B. der medizinisch-beruflichen Rehabilitation [5, 6]. Die Phase F bezeichnet die „funktionserhaltende Dauerpflege“ in spezialisierten Pflegeeinrichtungen, die u. a. über Expertise im Trachealkanülen- und Dysphagiemanagement verfügen [5]. Dieses Phasenmodell hat sich empirisch in Deutschland sehr bewährt.

Die Versorgungsrealität in der Bundesrepublik Deutschland stellt sich so dar, dass die NNFR als spezialisierte Leistung ganz überwiegend in neurologischen Facheinrichtungen, leistungsrechtlich als Krankenhausbehandlung, erbracht wird [7–9], dies allerdings oft ohne explizite Erwähnung in den Krankenhausplänen der Länder [9]. Als spezialisierte Krankenhausleistung kann die NNFR entweder unter Bedingungen des DRG-Systems (mit einer eigenen Prozedur: Operationen- und Prozedurenschlüssel [OPS] 8‑552 „neurologisch-neurochirurgische Frührehabilitation“; [10]) oder in einer „besonderen Einrichtung“ erbracht werden. Diese „besonderen Einrichtungen“ werden gemäß § 17b Abs. 1 Satz 15 KHG aus dem DRG-Vergütungssystem herausgenommen.

Eine kurze, repräsentative Umfrage der DGNR in diesem Jahr erfasste bereits 68 Weaningeinheiten in der NNFR, mit einer Gesamtsumme von 1094 Betten in Deutschland (Spannbreite 2 bis 68, Mittelwert 16,1 Betten pro Einheit; [11]).

Um die Strukturen der Beatmungsentwöhnung in der NNFR besser erfassen zu können, hat die Deutsche Gesellschaft für Neurorehabilitation (DGNR) eine detailliertere Umfrage durchgeführt, deren Ergebnisse in dieser Publikation dargestellt werden sollen.

## Methodik

Von der DGNR wurden im Februar 2020 insgesamt 381 (ärztliche) Mitglieder der Fachgesellschaft per E‑Mail angeschrieben mit der Bitte, dass die Einrichtungen, in denen sie tätig sind, soweit sie neurologische Weaningbetten vorhalten, sich an der Umfrage beteiligen. Die Teilnahme war für die Mitglieder bzw. Einrichtungen freiwillig. Von den über die Mitglieder kontaktierten Einrichtungen nahmen 36 an der Umfrage teil; in Bezug auf eine deutlich weniger umfangreiche und damit für die Befragten weniger aufwendige Umfrage [11], die bei gleichem Verteiler 68 Weaningeinheiten dokumentierte, entspricht dies einer Teilnahmequote von 53 %. Die erfassten Daten wurden mit dem Programm SPSS®, Version 26.0 (SPSS Inc., Chicago, USA) ausgewertet.

Die WennFrüh-Studie erhob Daten zu Strukturmerkmalen der Einrichtungen, u. a. die ärztliche Leitung, apparative und therapeutische Ausstattung betreffend, ferner für den Jahresberichtszeitraum 2019 aggregierte anonyme Daten zu Fallzahl, Behandlungsergebnis und Diagnosespektrum; schließlich wurden Angaben über die Auswirkungen der Pflegepersonaluntergrenzenverordnung, zu dem Wunsch nach Zertifizierung und der poststationären Versorgung beatmeter/trachealkanülierter Frührehabilitanden erfasst.

Die gemachten Angaben bezogen sich alle auf die jeweilige Einrichtung als Beobachtungseinheit und schlossen keine spezifischen Personen zuordenbare Angaben ein. Es wurden lediglich anonyme aggregierte Daten erhoben. Entsprechend bedurfte es – auch nach Einschätzung der Geschäftsführung der Ethikkommission der Universitätsmedizin Greifswald – keines Votums der Ethikkommission gemäß § 15 der Berufsordnung, da keine Forschung mit personenbezogenen Daten betrieben wurde.

## Ergebnisse

### Versorgungsstrukturen

Die 36 NNFR-Weaningeinheiten verteilten sich über 11 Bundesländer (Tab. 1) und umfassten insgesamt 496 Betten für die Beatmungsentwöhnung (Spannbreite 2 bis 44, Mittelwert 13,8 Betten pro Einrichtung, Standardabweichung 10,6). Bei den meisten Anbietern handelte es sich um Fachkrankenhäuser, die nach DRG (*n* = 13; 36,1 %) abrechneten oder über den Status der „besonderen Einrichtung“ (*n* = 9; 25,0 %) verfügten. Zehn Leistungserbringer (27,8 %) waren als Fachabteilung Teil eines Akutkrankenhauses, wobei es sich in 7 Fällen um ein Krankenhaus der Maximal- und in 3 Fällen um ein Krankenhaus der Regelversorgung handelte. In 2 Fällen wurde das Weaning in Rehabilitationskliniken ohne Krankenhausstatus durchgeführt und in weiteren 2 Fällen der leistungsrechtliche Status nicht genauer differenziert.

**Table 1:** 

Bundesland	Anzahl der Einheiten	Weaningbetten	Frührehaabteilung	Fachklinik (DRG)	Fachklinik (Besondere Einrichtung)	Rehabilitationsklinik	Andere Klinik
Baden-Württemberg	5	39	1	0	4	0	0
Bayern	2	22	0	1	0	0	1
Berlin	3	53	1	0	2	0	0
Brandenburg	3	67	0	1	2	0	0
Hessen	4	96	0	4	0	0	0
Mecklenburg-Vorpommern	1	12	0	1	0	0	0
Niedersachsen	8	82	5	2	0	1	0
Nordrhein-Westfalen	3	26	0	3	0	0	0
Rheinland-Pfalz	1	6	1	0	0	0	0
Sachsen	4	44	0	1	1	1	1
Schleswig-Holstein	2	49	2	0	0	0	0
*Summe*	*36*	*496*	*10*	*13*	*9*	*2*	*2*

Die meisten Weaningbetten (65,8 %) befanden sich erwartungsgemäß auf einer als „Intensivstation“ bezeichneten Einheit, während 29,3 % einer „Intermediate-care“-Station und nur 4,9 % dem Normalstationsbereich zugeordnet wurden. Mehrfachnennungen waren hier möglich.

Neben der NNFR boten die meisten Leistungserbringer auch noch die BAR-Phasen C (75,0 %) und D (69,4 %) an, wobei die eigenständige Akutversorgung (Phase A), z. B. auf einer Stroke-Unit, nur von 2 (5,6 %) und die Phase E von 3 (8,3 %) Studienteilnehmern berichtet wurde. Während der Beatmungsentwöhnung wurde die Prozedur „neurologisch-neurochirurgische Frührehabilitation“ (OPS 8‑552) in 33 von 36 Einheiten (91,7 %) erbracht.

### Fallzahl und Behandlungsergebnisse

Insgesamt 29 der 36 Einrichtungen teilten ihre Weaningfallzahl für den Jahresberichtszeitraum 2019 mit (aus einer Einrichtung wurden Daten aus 2018 mitgeteilt). Diese betrug kumulativ 3720 (Spannbreite 5 bis 534 Fälle im Erhebungsjahr, Mittelwert 128,3 Fälle pro Weaningeinheit, Standardabweichung 116,3).

Aus 20 Einrichtungen lagen vollständige Angaben zur Zahl der primär erfolgreich entwöhnten, der mit Heimbeatmung entlassenen und der verstorbenen Patienten des Berichtsjahres vor. Insgesamt handelte es sich um 2516 Fälle, von denen 2097 (83,3 %) primär erfolgreich geweant und nur 120 (4,8 %) mit Heimbeatmung entlassen werden mussten. Die Mortalität in dieser Stichprobe lag bei 11,0 % (*n* = 276).

Von 16 Studienteilnehmern wurde die Gesamtsumme der erbrachten Beatmungsstunden eines Jahres angegeben, es ergab sich eine Gesamtsumme von 1.172.855 Beatmungsstunden im Jahr 2019 (Spannbreite 9189 bis 226.236 Stunden, Mittelwert 73.303 Stunden pro Weaningeinheit, Standardabweichung 55.704).

### Diagnosespektrum

Die von 20 Kliniken zur Verfügung gestellte Diagnosenverteilung ist der Tab. 2 zu entnehmen. Führend ist die Diagnose der „critical illness polyneuropathy/myopathy“ (CIP/CIM), die etwa ein Viertel aller Fälle ausmachte (23,3 %), gefolgt vom ischämischen Schlaganfall (21,0 %), der intrazerebralen Blutung (17,7 %) und dem hypoxischen Hirnschaden (11,6 %). Diese vier führenden Diagnosegruppen machten drei Viertel aller Weaningfälle aus.

**Table 2:** 

Diagnosegruppe	(*n*)	(%)
„Critical illness polyneuropathy“ (CIP)	630	23,3
Hirninfarkt	568	21,0
Intrazerebrale Blutung	477	17,7
Hypoxischer Hirnschaden	312	11,6
Schädel-Hirn-Trauma	246	9,1
Querschnittlähmungen	148	5,5
Entzündliche Neuropathien (CIDP, GBS) und Polyneuropathien anderer Ursache	75	2,8
Polytrauma	56	2,1
Neuromuskuläre Erkrankungen (ALS; Muskeldystrophien)	41	1,5
Neoplasien	37	1,4
Andere Erkrankungen	110	4,1
*Summe*	*2700*	*100*

*ALS* Amyotrophe Lateralsklerose

### Ärztliche Kompetenz

Was die ärztliche Versorgung der Einheiten anbelangt, so wurden sie in 28 von 36 Fällen von einem Facharzt für Neurologie geführt (77,8 %). In 10 Weaningeinheiten (27,8 %) hatte ein Facharzt für Anästhesie die Leitung und in 4 Einheiten (11,1 %) ein Facharzt für Innere Medizin. Die Überschneidungen kamen dadurch zustande, dass es auch kollegiale Leitungsmodelle (Neurologe plus Anästhesist oder Neurologe plus Internist) gab.

Von den die Weaningeinheit leitenden Ärzten verfügten 27 (75,0 %) über die Zusatzbezeichnung „Intensivmedizin“, 20 leitende Ärzte hatten eine Weiterbildungsermächtigung für „Intensivmedizin“ (55,5 % aller teilnehmenden Kliniken), 9 davon für die Dauer von 6 Monaten, 3 für 12 Monate, einer für 18 und 7 Kollegen für die volle Weiterbildungszeit von 24 Monaten. Die ärztlichen Leiter ohne Zusatzbezeichnung „Intensivmedizin“ gaben an, dass sie nach der Facharztweiterbildung im Mittel 15,9 Jahre (Streubreite 2 bis 28, Standardabweichung 7,8) praktische Erfahrung in der Intensivmedizin bzw. der Beatmungsentwöhnung neurologisch-neurochirurgischer Frührehabilitanden gesammelt hatten.

Pneumologische Kompetenz war in 17 Kliniken (47,2 %) verfügbar, und zwar in 7 Fällen durch Präsenz auf der Station, in 4 Fällen durch ein internes und in 6 Fällen durch ein externes Konsil. Die Nutzung der pneumologischen Kompetenz ist in Tab. 3 wiedergegeben.

**Table 3:** 

Nutzungsfrequenz	(*n*)	(%)
Täglich	5	29,4
Mehr als 4‑mal im Monat	1	5,9
4‑mal im Monat	1	5,9
2‑mal im Monat	4	23,5
1‑mal im Monat	3	17,6
Nie	3	17,6
*Summe*	*17*	*100*

### Apparative und therapeutische Ausstattung

Die apparative und therapeutische Ausstattung der NNFR-Weaningeinheiten ist Tab. 4 zu entnehmen. 100 % der Teilnehmer verfügten über eine Bronchoskopie und eine Blutgasanalyse. Der therapeutische Schwerpunkt in der NNFR zeigt sich in einer hohen Verfügbarkeit von Physiotherapie, Ergotherapie, Logopädie und differenzierter Dysphagietherapie. Bemerkenswert ist die Vorhaltung von Atmungstherapie in 86 % der Einrichtungen.

**Table 4:** 

Gerät/Therapieform	(*n*)	(%)
Oszillierende und nichtoszillierenden PEP-Systeme	23	63,9
Mechanische Insufflatoren-Exsufflatoren	29	80,6
Bronchoskopie	36	100
Atmungsfunktionstests, z. B. „peak expiratory flow“, „cough peak flow“, Bed-side-Spirometrie, Rapid Shallow Breathing Index, Cuffl-leak-Test	28	77,8
Dilatative Tracheotomie	21	58,3
Erhebung eines Sedierungsscores (z. B. Richmond Agitation-Sedation Scale)	27	75,0
Blutgasanalyse (arteriell/kapillär)	36	100
Atmungstherapie (z. B. Deutsche Gesellschaft für Pneumologie oder Deutsche Gesellschaft für pflegerische Weiterbildung)	31	86,1
Physiotherapie	35	97,2
Ergotherapie	35	97,2
Logopädie	36	100
Dysphagietherapie	35	97,2
FEES	34	94,4
Musiktherapie	16	44,4

*FEES* Flexible Endoskopische Evaluation des Schluckens

### Auswirkungen der Pflegepersonaluntergrenzenverordnung

Insgesamt 24 von 36 Teilnehmern gaben an, die Untergrenzen für die Intensivstation (Besetzung Tagschicht 1:2,5 bzw. ab 2021 1:2) zugrunde zu legen. 19 davon (79,2 %) gaben an, dass sie die Grenzen zu Beginn des Jahres 2020 (vor der SARS-CoV-2-Pandemie) überwiegend hätten einhalten können, dazu mussten allerdings 18 Kliniken Betten sperren (75 %), 6 waren bereit, Sanktionen in Kauf zu nehmen (25 %) und 13 (54,2 %) mussten auf die Dienstleistungen von Zeitarbeitsfirmen zurückgreifen. Hier waren Mehrfachantworten möglich.

### Zertifizierung von Weaningeinheiten in der NNFR

Die Einführung einer Zertifizierung für neurologische Weaningeinheiten begrüßten 22 Studienteilnehmer (61,1 %), während 6 (16,7 %) das ablehnten und 8 (22,2 %) unentschlossen waren.

### Poststationäre Versorgung

Insgesamt 24 Weaningeinheiten (66,7 %) boten eine ambulante Nachbetreuung von beatmet oder trachealkanüliert entlassenen Frührehabilitanden an, 2 (5,6 %) eine stationäre Wiederaufnahme und 2 (5,6 %) eine pneumologische Nachversorgung. Damit hatten immerhin 77,8 % der Weaningeinheiten ein ambulantes oder stationäres Versorgungskonzept für diese Patienten.

## Diskussion

In Anbetracht von über 1000 Betten in 68 deutschen Weaningeinheiten der NNFR [11] zeigt die Teilnahmequote der WennFrüh-Studie mit 36 Teilnehmern und ca. 500 Betten an, dass sich vermutlich nur etwa die Hälfte der Leistungserbringer an der Umfrage beteiligt hat. Dennoch können detaillierte Strukturdaten aus immerhin 11 Bundesländern in dieser Publikation berichtet werden.

In mehr als der Hälfte der Fälle waren die Weaningeinheiten Bestandteil neurologischer Fachkrankenhäuser, die ihre Leistungen zumeist im DRG-System oder als besondere Einrichtung erbrachten. Nur etwa ein Drittel der Einheiten war Teil einer Fachabteilung für NNFR in einem Akutkrankenhaus, zumeist in einem Zentrum der Maximalversorgung. Nur 2 Leistungsanbieter hatten keinen Status als Krankenhaus, sondern den einer Rehabilitationseinrichtung (§ 40 SGB V).

In den meisten Häusern gab es neben der NNFR (inklusive Weaning) auch noch anschließende Rehabilitationsangebote in den BAR-Phasen C (75,0 %) und D (69,4 %), sodass ein phasenübergreifendes und nahtloses Versorgungsangebot vorlag.

Bemerkenswert ist, dass während der Beatmungsentwöhnung die neurologisch-neurochirurgische Frührehabilitationsprozedur (OPS 8‑552) von über 90 % der teilnehmenden Kliniken erbracht wurde. Mit der Forderung, 300 min Therapie pro Tag im Durchschnitt der Behandlung zu erbringen, stellt dies bei beatmeten Patienten nicht nur eine Herausforderung für die Klinik, sondern auch für die nicht immer klinisch stabilen Frührehabilitanden dar. Auch unter ökonomischen Gesichtspunkten ist dieser Sachverhalt relevant, denn bei einer Beatmungs-DRG wirkt die Kodierung des OPS 8‑552 nicht erhöhend auf das Relativgewicht. Trotz des hieraus resultierenden Fehlanreizes, diese Leistung nicht zu erbringen, führen die Einrichtungen der NNFR – dies belegt die WennFrüh-Studie – diese regelhaft durch. Somit verdeutlicht die durch WennFrüh ermittelte konsequente Durchführung des OPS 8‑552 bei Weaningpatienten durch die Einrichtungen der NNFR die medizinische Notwendigkeit, auch bei dieser Klientel die intensive interprofessionelle Frührehabilitation als integralen Bestandteil der Behandlung durchzuführen. Diese Notwendigkeit der intensiven interprofessionellen Frührehabilitation bestätigt wiederum die Empfehlung der S2k-Leitlinie zum prolongierten Weaning in der NNFR [2], dass beatmete Patienten mit Erkrankungen des zentralen und/oder peripheren Nervensystems und/oder (neuro-)muskulären Erkrankungen so früh wie möglich in eine neurologisch-neurochirurgische Frührehabilitationseinrichtung mit intensivmedizinischer und Weaningkompetenz verlegt werden sollen. Denn außerhalb dieser Strukturen können diese regelhaft notwendigen Leistungen nicht erbracht und damit die Betroffenen nicht adäquat behandelt werden.

Insgesamt wurden die Behandlungsergebnisse von immerhin 2516 Beatmungsfällen berichtet, von denen über 80 % primär erfolgreich entwöhnt und weniger als 5 % mit Heimbeatmung entlassen werden mussten. Die Mortalität in dieser Stichprobe lag bei 11,0 %. Vergleicht man diese Ergebnisse mit Angaben aus der Literatur, so wirken sie plausibel. Der Anteil primär erfolgreich entwöhnter Patienten in der NNFR wurde in verschiedenen Studien zwischen 68,3 und 92,3 % angegeben [1, 12, 13]. Die Mortalität lag zwischen 6,1 und 16,6 % [1, 12, 13]. Auch der Anteil der mit Heimbeatmung entlassenen Patienten (ca. 5 %) erscheint vor dem Hintergrund der Angaben in der Literatur nachvollziehbar [1, 12, 13]. Die Zahlen bestätigten die hohe Leistungsfähigkeit und Effektivität der NNFR in Bezug auf ein erfolgreiches Weaning und die Vermeidung außerklinischer Beatmungsnotwendigkeit. Auch diese Beobachtung legt nahe, dass das Klientel mit prolongiertem Weaningbedarf bei neurologischen Erkrankungen sehr stark von einer Behandlung in Einrichtungen der NNFR profitiert.

Im Diagnosespektrum ist die Diagnose der „critical illness polyneuropathy/myopathy“ (CIP/CIM) führend, die etwa ein Viertel aller Fälle ausmachte (23,3 %). Dieser Anteil ist etwas geringer als in einer großen multizentrischen Untersuchung, in der immerhin jeder 3. Patient mit dieser Aufnahmediagnose behandelt wurde [1]. Nicht selten wird vermutet, dass es sich bei einer CIP um eine „Feigenblattdiagnose“ handele, die eine primäre Fehlbelegung in einer neurologischen Klinik verhindern helfen solle. Tatsächlich konnte aber in einer Studie gezeigt werden, dass sie bei 83 % der Patienten klinisch bzw. klinisch-neurophysiologisch evident und mit einem erheblichen Behandlungsaufwand bei sehr schwer kranken, multimorbiden Patienten verbunden ist [14].

Besonderes Augenmerk hat die WennFrüh-Studie auf die ärztliche Kompetenz gerichtet, da insbesondere die Weiterbildungsmöglichkeiten für Neurologen im Hinblick auf die Zusatzbezeichnung „Intensivmedizin“ begrenzt sind [15]. Aus diesem Grund wurde die in der pneumologischen Weaningleitlinie geforderte formale intensivmedizinische Qualifikation des ärztlichen Leiters [3] nicht in die DGNR-Leitlinie aufgenommen [2]. In letzterer wird die Qualifikation des Behandlungsteams mit einem Fokus auf Erfahrung wie folgt definiert: „Idealerweise sollten geeignete Einrichtungen über ein Behandlungsteam mit langjähriger intensivmedizinischer, neurologischer und neurochirurgischer Expertise verfügen und von einer/einem Fachärztin/Facharzt für Neurologie oder Neurochirurgie mit intensivmedizinischer Erfahrung geleitet werden.“ [2]. Die WennFrüh-Studie zeigte zwar, dass 75 % der ärztlichen Leiter über die Zusatzbezeichnung „Intensivmedizin“ verfügten, die Weiterbildungsmöglichkeiten innerhalb der Einrichtungen, die bei WennFrüh teilgenommen haben, waren aber sehr eingeschränkt. Nur in 7 Einrichtungen (19,4 %) lag die volle Weiterbildungsermächtigung für „Intensivmedizin“ (24 Monate) vor. Die ärztlichen Leiter von Weaningbereichen der NNFR, welche nicht die Zusatzbezeichnung „Intensivmedizin“ besaßen, verfügten allerdings in hohem Maße über die von der DGNR-Leitlinie geforderte „intensivmedizinische Erfahrung“: Ihre Erfahrung in der Intensivmedizin bzw. der Beatmungsentwöhnung neurologisch-neurochirurgischer Frührehabilitanden erstreckte sich nämlich im Mittel über etwa 16 Jahre. Dieses unterstützt die in der Leitlinie „Prolongiertes Weaning“ dokumentierte Position der DGNR, dass die Forderung nach der Zusatzbezeichnung „Intensivmedizin“ als formales Kriterium für ärztliche Leiter von Weaningbereichen in der NNFR nicht sinnvoll ist.

Die für pneumologische Weaningzentren eingeforderte pneumologische Kompetenz war nur in weniger als der Hälfte der teilnehmenden Einrichtungen verfügbar, wurde dann aber in zwei Dritteln der Einrichtungen nur bis zu 4‑mal im Monat genutzt. Diese geringe Nutzungsfrequenz bei guten Behandlungsergebnissen spricht dafür, dass angesichts der spezifischen Patientenklientel der NNFR und dem daraus resultierenden frührehabilitativen Behandlungsschwerpunkt eine ständige pneumologische Präsenz verzichtbar ist. Die Bedeutung der Zusammenarbeit der medizinischen Fachdisziplinen bei der Behandlung multimorbider, beatmeter Patienten war nicht Gegenstand von WennFrüh, sollte aber in zukünftigen Untersuchungen berücksichtigt werden.

Der besondere therapeutische Schwerpunkt in der NNFR zeigt sich in einer hohen Verfügbarkeit von Physiotherapie, Ergotherapie, Logopädie und differenzierter Dysphagietherapie. Obwohl die Kodierung des OPS 8‑552 nicht zu einem höheren Erlös führt (s. oben), haben über 90 % eine neurologisch-neurochirurgische Frührehabilitation nach den Regeln der Prozedur durchgeführt und diese therapeutischen Ressourcen „an das Bett gebracht“. Dies trifft auch für die relativ neue Disziplin der „Atmungstherapie“ zu [16], einer Weiterbildung, die primär für (Intensiv‑)Pflegekräfte vorgesehen ist und von 86 % der Leistungsanbieter vorgehalten wurde.

Die WennFrüh-Studie lässt auch Aussagen über die Auswirkungen der Pflegepersonaluntergrenzenverordnung (PpUGV) zu. Die meisten Kliniken legten die Untergrenzen für eine Intensivstation zugrunde. Zwar konnten die Untergrenzen vor der SARS-CoV-2-Pandemie weitestgehend eingehalten werden, aber nur zum Preis von Kapazitätseinbußen. Drei Viertel der Leistungserbringer waren gezwungen, Betten zu schließen, um die Untergrenzen einhalten zu können. Dies ist für die Versorgung schwerstbetroffener Frührehabilitanden ein besorgniserregender Befund, zumal bereits heute die Behandlungskapazitäten in der NNFR, v. a. auf Weaningstationen, gar nicht ausreichen. In einer Studie konnte gezeigt werden, dass nur 45 % der niedersächsischen Patienten im eigenen Bundesland einen Frührehabilitationsplatz erhalten, bei den Beatmeten waren es sogar nur 37 % [8]. Andererseits ist das Anliegen der PpUGV, so berechtigt es dem Grunde nach ist, durch die PpUGV für die NNFR nicht sachgerecht umgesetzt. Denn in der NNFR wird der Patient von einem interprofessionellen Team betreut, in dem auch therapeutische Berufsgruppen in erheblichem Umfang Leistungen erbringen, die sonst auf einer Intensivstation allein durch Pflegepersonal erbracht wird, wie z. B. Körperpflege mit therapeutischem Ansatz, Mobilisation und Trachealkanülenmangement. Doch diese Aspekte der Patientenversorgung finden in der PpUGV keine Berücksichtigung. Damit generiert die PpUGV in der NNFR teilweise indirekt eine Kapazitätsverknappung (Nichtnutzung von Bettenkapazitäten), obwohl keine konkrete Patientenmangelversorgung besteht. Hier gibt es Anpassungsbedarf, um eine ausreichende Versorgung schwerst Betroffener zu ermöglichen und damit Lebensqualität zu fördern und die Vermeidung langfristiger außerklinischer Beatmung zu garantieren. Sollte die durch PpUGV herbeigeführte Kapazitätsverknappung in der NNFR aufrechterhalten werden, sind nicht geringe Folgekosten zu befürchten, da mehr Patienten, die geweant und dekanüliert werden könnten, stattdessen ggf. direkt von Akutintensivstationen in die außerklinische Intensivpflege verlegt werden müssten. Dadurch würde in der außerklinischen Intensivpflege noch mehr Pflegepersonal gebunden, wodurch sich der Pflegepersonalmangel in den Krankenhäusern weiter verschlechtern könnte.

Was die in der Pneumologie bereits durchgeführte Zertifizierung von Weaningzentren anbelangt, sprachen sich immerhin 61 % der Studienteilnehmer dafür aus, dies auch für Weaningzentren in der NNFR vorzusehen. In Anbetracht der Versorgungsrelevanz und des spezifischen Behandlungsansatzes kann die Etablierung einer Zertifizierung für Weaningzentren in der NNFR ein relevantes Thema für die DGNR sein, um Qualitätssicherung in diesem Versorgungsbereich zu unterstützen.

Der Forderung der Leitlinie zum prolongierten Weaning in der NNFR nach einer poststationären Versorgung [2] ist bereits teilweise erfüllt, da immerhin zwei Drittel der neurologischen Weaningeinheiten eine ambulante Nachbetreuung von beatmet oder trachealkanüliert entlassenen Frührehabilitanden anboten. In 2 Fällen (5,6 %) beinhaltete das Nachsorgekonzept eine stationäre Wiederaufnahme.

## Ausblick und Herausforderungen

In der Summe zeigt die WennFrüh-Studie, dass die in Deutschland seit Jahrzehnten etablierte NNFR auch ein spezialisiertes Behandlungsangebot für Frührehabilitanden im prolongierten Weaning anbietet. Weitere Studien mit einer höheren Teilnehmerzahl sind wünschenswert, um detailliertere Einblicke in diese hoch spezialisierte Versorgung zu ermöglichen.

Es ist evident, dass das Weaning in der NNFR in der Versorgung schwerstkranker neurologischer und neurochirurgischer Patienten in Deutschland einen wichtigen und sehr effektiven Baustein darstellt.

In der Definition von neuen OPS-Ziffern sollte die DGNR daher eingebunden werden, um die Besonderheiten des prolongierten Weanings in diesem Bereich zu vertreten und deutlich zu machen. Unter anderem zeigen die erhobenen Daten deutlich, dass Weaning und intensive interprofessionelle Frührehabilitation regelhaft notwendige integrale Bestandteile der Behandlung dieser Patienten sind, ein Sachverhalt der derzeit im DRG-System nicht abgebildet ist. Die Behandlungsleitung muss entsprechend der Leitlinie der DGNR in Händen kompetenter und erfahrener Fachärzte liegen, jedoch nicht formal an der Zusatzbezeichnung Intensivmedizin ausgerichtet sein. Auch sind Verordnungen, die die intensivmedizinische Versorgung insgesamt betreffen, wie die PpUGV für die NNFR nicht genügend differenziert und können sich auf die Versorgung nachteilig auswirken.

## Fazit für die Praxis

- Weaning in der NNFR stellt in der Versorgung schwerstkranker neurologischer und neurochirurgischer Patienten einen wichtigen und sehr effektiven Baustein dar.
- Schwerstkranke neurologische und neurochirurgische Patienten im prolongierten Weaning bedürfen regelhaft der intensiven interprofessionellen Frührehabilitation.
- Der Operationen- und Prozedurenschlüssel (OPS) bedarf der Weiterentwicklung, um das Weaning in der NNFR adäquat abzubilden.
- Die Pflegepersonaluntergrenzenverordnung (PpUGV) könnte – entgegen ihrem Anliegen – Versorgung in der NNFR verknappen und bedarf für die NNFR einer Anpassung.

## Abkürzungen

ALS: Amyotrophe Lateralsklerose
BAR: Bundesarbeitsgemeinschaft für Rehabilitation
CIDP: Chronisch-inflammatorische demyelinisierende Polyneuritis
CIP/CIM: Critical illness polyneuropathy/myopathy
DGNR: Deutsche Gesellschaft für Neurorehabilitation
DGP: Deutsche Gesellschaft für Pneumologie
DGpW: Deutsche Gesellschaft für pflegerische Weiterbildung
DRG: Diagnosis related groups
FEES: Flexible endoskopische Evaluation des Schluckens
GBS: Guillain-Barre-Syndrom
NIV: Non-invasive ventilation
NNFR: Neurologisch-neurochirurgische Frührehabilitation
OPS: Operationen- und Prozedurenschlüssel
PEP: Positive expiratory pressure
PpUGV: Pflegepersonaluntergrenzenverordnung
SARS-CoV‑2: Severe acute respiratory syndrome coronavirus 2
SGB: Sozialgesetzbuch

## References

[1] Oehmichen F, Ketter G, Mertl-Rötzer M (2012). Beatmungsentwöhnung in neurologischen Weaningzentren. Eine Bestandsaufnahme der AG Neurologische Frührehabilitation. Nervenarzt.

[2] Rollnik JD, Adolphsen J, Bauer J (2017). Prolongiertes Weaning in der neurologisch-neurochirurgischen Frührehabilitation. S2k-Leitlinie herausgegeben von der Weaning-Kommission der Deutschen Gesellschaft für Neurorehabilitation e. V. (DGNR). Nervenarzt.

[3] Schönhofer B, Geiseler J, Dellweg D (2019). Prolongiertes Weaning. S2k-Leitlinie herausgegeben von der Deutschen Gesellschaft für Pneumologie und Beatmungsmedizin e. V. Pneumologie.

[4] Musicco M, Emberti L, Nappi G (2003). Early and long-term outcome of rehabilitation in stroke patients: the role of patient characteristics, time of initiation, and duration of interventions. Arch Phys Med Rehabil.

[5] Bundesarbeitsgemeinschaft für Rehabilitation (1995). Bundesarbeitsgemeinschaft für Rehabilitation. Empfehlungen zur Neurologischen Rehabilitation von Patienten mit schweren und schwersten Hirnschädigungen in den Phasen B und C.

[6] Rollnik JD, Platz T, Böhm KD (2013). Die medizinisch-berufliche Rehabilitation in der Neurologie. Akt Neurol.

[7] Stier-Jarmer M, Koenig E, Stucki G (2002). Strukturen der neurologischen Frührehabilitation (Phase B) in Deutschland. Phys. Med. Rehabil. Kurort.

[8] Roesner M, Beyer J, Dohm PC (2019). Neurologisch-neurochirurgische Frührehabilitation im Land Niedersachsen und Bremen. Fortschr Neurol Psychiatr.

[9] Rollnik JD, Platz T, Böhm KD (2011). Argumente für eine Zuordnung der neurologisch-neurochirurgischen Frührehabilitation (Phase B) zum Krankenhausbereich (§ 39 SGB V). Positionspapier der Kliniken des BDH Bundesverband Rehabilitation. Akt Neurol.

[10] Rollnik JD, Janosch U (2010). Verweildauerentwicklung in der neurologischen Frührehabilitation. Dtsch Arztebl Int.

[11] Platz T, Bender A, Dohle C, Gorsler A, Knecht S, Liepert J, Mokrusch T, Sailer M (2020). German hospital capacities for prolonged mechanical ventilator weaning in neurorehabilitation—results of a representative survey. Neurol Res Pract.

[12] Rollnik JD, Berlinghof K, Lenz O, Bertomeu A (2010). Beatmung in der neurologischen Frührehabilitation. Akt Neurol.

[13] Rollnik JD, Krauss JK, Gutenbrunner C (2017). Weaning of neurological early rehabilitation patients from mechanical ventilation: a retrospective observational study. Eur J Phys Rehabil Med.

[14] Schmidt SB, Rollnik JD (2016). Critical illness polyneuropathy (CIP) in neurological early rehabilitation: clinical and neurophysiological features. BMC Neurol.

[15] Busse O, Hillmann S, Grond M (2018). Neurointensivmedizin in Deutschland. Nervenarzt.

[16] Schmidt SB, Reck C, Boltzmann M, Rollnik JD (2019). Einfluss der Atmungstherapie auf die Inzidenz von nosokomialen Pneumonien in der neurologisch-neurochirurgischen Frührehabilitation: Ergebnisse einer Fall-Kontroll-Analyse. Rehabilitation.

